# Transcriptional regulation of the grape cytochrome P450 monooxygenase gene CYP736B expression in response to *Xylella fastidiosa *infection

**DOI:** 10.1186/1471-2229-10-135

**Published:** 2010-07-01

**Authors:** Davis W Cheng, Hong Lin, Yuri Takahashi, M Andrew Walker, Edwin L Civerolo, Drake C Stenger

**Affiliations:** 1 San Joaquin Valley Agricultural Science Center, USDA-ARS 9611 South Riverbend Avenue, Parlier, CA 93648, USA; 2 Department of Biology, California State University, Fresno, CA 93740, USA; 3 Department of Viticulture and Enology, University of California, Davis, CA 95616, USA; 4 Department of Food sciences, Ehime Women's College, Uwajima, Ehime, 798-0025 Japan

## Abstract

**Background:**

Plant cytochrome P450 monooxygenases (CYP) mediate synthesis and metabolism of many physiologically important primary and secondary compounds that are related to plant defense against a range of pathogenic microbes and insects. To determine if cytochrome P450 monooxygenases are involved in defense response to *Xylella fastidiosa *(*Xf*) infection, we investigated expression and regulatory mechanisms of the cytochrome P450 monooxygenase *CYP736B *gene in both disease resistant and susceptible grapevines.

**Results:**

Cloning of genomic DNA and cDNA revealed that the *CYP736B *gene was composed of two exons and one intron with GT as a donor site and AG as an acceptor site. *CYP736B *transcript was up-regulated in PD-resistant plants and down-regulated in PD-susceptible plants 6 weeks after *Xf *inoculation. However, *CYP736B *expression was very low in stem tissues at all evaluated time points. 5'RACE and 3'RACE sequence analyses revealed that there were three candidate transcription start sites (TSS) in the upstream region and three candidate polyadenylation (PolyA) sites in the downstream region of *CYP736B*. Usage frequencies of each transcription initiation site and each polyadenylation site varied depending on plant genotype, developmental stage, tissue, and treatment. These results demonstrate that expression of *CYP736B *is regulated developmentally and in response to *Xf *infection at both transcriptional and post-transcriptional levels. Multiple transcription start and polyadenylation sites contribute to regulation of *CYP736B *expression.

**Conclusions:**

This report provides evidence that the cytochrome P450 monooxygenase *CYP736B *gene is involved in defense response at a specific stage of *Xf *infection in grapevines; multiple transcription initiation and polyadenylation sites exist for *CYP736B *in grapevine; and coordinative and selective use of transcription initiation and polyadenylation sites play an important role in regulation of *CYP736B *expression during growth, development and response to *Xf *infection.

## Background

Plant cytochrome P450 monooxygenases are a group of redox proteins that catalyze various oxidative reactions [1]. It is proposed that cytochrome P450 monooxygenases mediate synthesis and metabolism of many physiologically important compounds. Steroids, fatty acids, lignins, terpenes, alkaloids, phenylpropanoids, and phytoalexins are examples of these primary and secondary compounds that act as plant defense agents against a range of diverse pathogenic microbes and insect pests [2-4]. Cytochrome P450 enzymes are also involved in several biosynthesis pathways in leaf tissues of *Arabidopsis *and function as part of the highly sophisticated network of plant defense reactions [5]. These defense responses include the hypersensitive response [6] and inhibition of growth of particular plant pathogens [7]. Gene expression analysis has revealed that most cytochrome P450 genes are strictly regulated in response to phytohormones (salicylic acid, jasmonic acid, ethylene and abscisic acid), pathogens (necrotrophic fungal pathogens *Alternaria alternata *and *A. brassicicola*), UV damage, heavy metal toxicity, mechanical injury, drought, high salinity and low temperatures [8]. Therefore, cytochrome P450 genes may be involved in plant defense responses to abiotic and biotic stresses. How cytochrome P450 genes are regulated in response to these stresses, especially to bacterial infection, is not understood.

The most widely cultivated grape species is *Vitis vinifera*. These grapevines are highly susceptible to many pests and pathogens, including *Xylella fastidiosa *(*Xf*), the bacterium responsible for Pierce's disease (PD). *Xf *is transmitted by xylem-feeding sharpshooters. PD has been an important but localized disease in California for over 100 years. Recently, the introduction of the glassy-winged sharpshooter vector into California resulted in new and range expanding epidemics of PD [9]. PD susceptible grapevines infected with *Xf *exhibit inhibited periderm development in stems (green islands), leaf blade separation from the petiole (matchsticks), irregular leaf scorching, fruit cluster dehydration, stunting and eventual plant death [10-12]. While cultivars of *V. vinefera *are susceptible to PD, several species of grapevine are resistant. A breeding program based on resistance from *V. arizonica *has been developed and led to genetic mapping of a single locus for resistance (*PdR1*) in the 9621 population [11,13]. This population is a cross of two half-sib genotypes, D8909-15 (*V. rupestris *'A. de Serres × *V. arizonica *b42-26) × F8909-17 (*V. rupestris *'A. de Serres' × *V. arizonica/candicans *b43-17) [13]. Two progeny from this population, one resistant (9621-67) and the other susceptible (9621-94), were characterized for transcriptomic profiles [14], which implicated involvement of cytochrome P450 genes in a defense response to *Xf *infection http://cropdisease.ars.usda.gov/vitis_at/main-page.htm.

To further characterize how cytochrome P450 monooxygenase genes are regulated in response to *Xf *infection, the genotypes 9621-67 and 9621-94 were used to study temporal and spatial expression and transcriptional regulation in inoculated greenhouse-grown plants. This report characterizes the structure, expression and transcript maturation of a cytochrome P450 monooxygenase encoded by the *CYP736 *gene.

## Results

### Genomic organization and structure of CYP736 genes in grape

**Figure 1:**
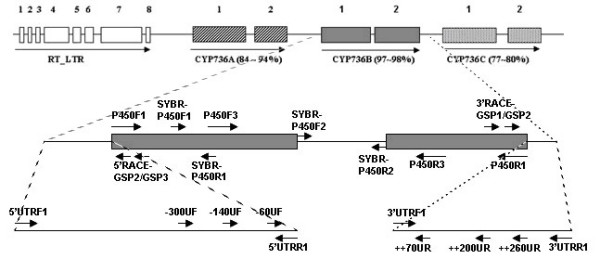
**Genomic organization and structure of cytochrome P450 *CYP736 *genes in grapevines**. There are three cytochrome P450 genes, *CYP736A*, *CYP736B *and *CYP736C*, flanked on the left with a RE-LTR gene on grape chromosome 7. Numbers on the top indicate order of exons, and the arrow indicates direction of transcription. Exon size and distance between genes and exons were drawn to approximate scale. The DNA coding sequence identity of each *CYP736 *gene derived from a large genomic contig CU459237.1 (4,713,370 bp) was compared with the target *CYP736B *gene cloned from PD- resistant 9621-67 grape selection and is shown below each gene member. The directions of PCR primers are shown in arrows at specific locations.

**Figure 2:**
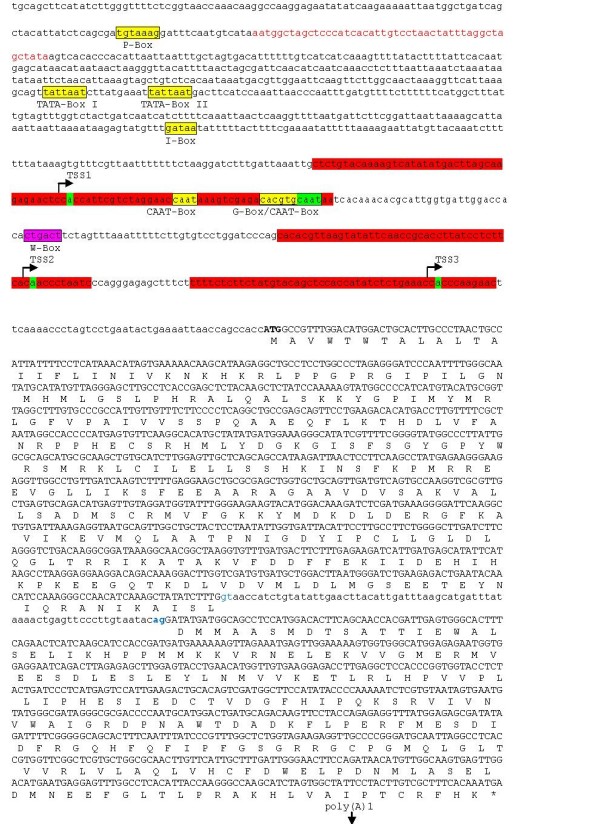
**Molecular structure and functional regulatory domains of grape *CYP736B***. A representative genomic DNA sequence from the PD- resistant selection 9621-67 was used as an example. The 5' upstream region, intron and 3' down-stream region are in lower case, exons are in upper case. Several regulatory elements were boxed with highlight in yellow in the 5'UTR-promoter region. The DNA fragment in red within the 5'UTR-promoter region was not present in PD- susceptible selection 9621-94. The transcription initiation regions are highlighted with red and the specific transcription start sites (TSS1, TSS2, and TSS3) in green are indicated with stem-arrows to indicate the direction of transcription. Three Poly(A) signal motifs were boxed with highlight in yellow and specific poly(A) sites, poly(A)1, poly(A)2 and poly(A)3, were highlighted in green and indicated by vertical arrows at 3'UTR. The DNA fragment in red at 3'UTR was not present in PD- resistant plants. The translation start codon was marked in bold and stop codon was marked with star (*). The donor site (gt) and receptor site (ag) for pre-mRNA splicing are marked in blue.

Genomic DNA sequences of grape cytochrome P450 monooxygenase genes cloned from PD- resistant and susceptible grapevines using the PCR primer P450F1 and P450R1 (Table 1) were 2,927 bp (Accession FJ620897) and 2,892 bp (Accession FJ620898), respectively. The cDNA sequences of grape cytochrome P450 monooxygenase genes cloned from leaf tissues of control PD-resistant and -susceptible grapevines using the PCR primers P450F1 and P450R1 (Table 1) were 1488 bp (Accessions FJ828517 and FJ828518). BLAST analysis of grapevine genomic DNA sequences revealed that the grape P450 monooxygenase gene has three homologous copies (*CYP736A*, *CYP736B *and *CYP736C*) in tandem on grape chromosome 7 (Figure 1). These three copies share 89%, 98% and 78% nucleotide sequence identity, respectively, compared to the closest grape genomic DNA contig sequence (Accession AM475392.1). The grape P450 monooxygenase genes we cloned from PD-resistant and susceptible grapevines were *CYP736B *genes that shared 93.6%, 99%, 98.6%, 90.3% genomic DNA sequence identity with each other in the 5' untranslated, exon, intron, and 3' untranslated regions (UTR), respectively. The grape P450 monooxygenase *CYP736B *genes cloned from PD- resistant and -susceptible grapevines have the same structure. Main features of these two *CYP736B *genes are: two exons, one intron with GT as 5'splice site and AG as 3' splice site. Compared to the *CYP736B *gene sequence from the PD-resistant grapevine, there are three C to T substitutions and one insertion (CTAT) in the upstream UTR region (1000 bp), one A to G and one T to C substitution in the exon 1 region, one G to A, one T to G and one C to G substitution in the intron region, one C to A, one A to C, one C to T, and one T to C substitution and a large deletion (39 bp) in the downstream non-coding region (300 bp) of the *CYP736B *gene from the PD-susceptible grapevine. DNA motif analysis indicated that there are nine major transcriptional regulatory motifs in the upstream non-coding region of the *CYP736B *gene, including one TATA box, five CAAT box, one I box, one G box, and one W box (Figure 2).

**Table 1:** PCR Primers used in this study

Name	Sequences (5' - 3')	Comments
P450F1	TGAAAATTAACCAGCCACCAT	Gene and cDNA cloning
P450R1	TTATATCATTTGTGAAAGC GACAAG	Gene and cDNA cloning
P450F2	TGCATGGACTGATGCAGAC	Expression detection in gel
P450R2	CTCCTCATTCATGTCCAACTC	Expression detection in gel
P450F3	TGGAGTTGCTCAGCAGCCATA	Internal primer for DNA sequencing
P450R3	TATATGGAA GCCATCGACTGTG	Internal primer for DNA sequencing
5'UTRF1	TGCAGCTTCATATCTTGGGTTTTCTC	5'UTR cloning (1000 bp)
5'UTRR3	GGTGGCTGGTT AATTTTCAGTATTCAG	5'UTR cloning (1000 bp)
3'UTRF1	ATATAATCTCTGTATCATTGCCAACTGAG	3'UTR cloning (600 bp)
3'UTRR1	ATGCTAAACATCAAATCGAATACTCTCA	3'UTR cloning (600 bp)
SYBR-P450F1	TGCATGGACTGATGCAGAC	Real-time qPCR for gene expression
SYBR-P450R1	CTCCTCATTCATGTCCAACTC	Real-time qPCR for gene expression
SYBR-P450F2	CCAACATCAAAGCTATATCTTTG	Real-time qPCR for pre-mRNA splicing
SYBR-P450R2	TGGAGGCTGCCATCAT ATC	Real-time qPCR for pre-mRNA splicing
P450-5'RACE GSP2	CGATACTTTTTGGATAGAGCTTGTAGAGC	First 5'RACE
P450-5'RACE GSP3	CGACAAGCTCCCTAACATATGCATATT	Second 5'RACE
P450-3'RACE GSP1	CGAGCAACTTGTTCATTGCTTTGAT	First 3'RACE
P450-3'RACE GSP2	CGAAGA TAACATGTTGGCAAGTGAGTT	Second 3'RACE
SYBR-P450B 5'UTRF1	ACTGACTTCTAGTTTAAATTTTTCTT	Real-time qPCR for 5'RACE
SYBR-P450B 5'UTRR1	TGGCTGGTTAATTTTCAGTATT	Real-time qPCR for 5'RACE
SYBR-P450B 3'UTRF1	ATATAATCTCTGTATC ATTGCCAA	Real-time qPCR for 3'RACE
SYBR-P450B 3'UTRR1	ACTACCAGATGAAAATCATTAAAT	Real-time qPCR for 3'RACE
P450-5'RACE GSP3	CGACAAGCTCCCTAACATATGCATATT	5' primer extension
P450-3'RACE GSP2	CGAAGA TAACATGTTGGCAAGTGAGTT	3' primer extension
-300UF	AGTCATATATGACTTAGCAAGAGAACTCCAC	PCR for pre-mRNA splicing at -300 TSS
-140UF	AACCGCACCTTATC CTCTTCACAA	PCR for pre-mRNA splicing at -140 TSS
-60UF	TGAAACCACCCAAGAACTTCAAAAC	PCR for pre-mRNA splicing at -60 TSS
++70UR	CATAAAGCATAAAGCGTTATTATTCATATTT	PCR for pre-mRNA splicing at ++70 poly(A) site
++200UR	CTAAGTTCCATATTCT TCCTTCTTAAGTTCA	PCR for pre-mRNA splicing at ++200 poly(A) site
++260UR	ACTCAGAACTAAGTTTTATTTCACTTGATCAA	PCR for pre-mRNA splicing at ++260 poly(A) site

### Temporal and spatial expression of CYP736B genes in grape

Expression of *CYP736B *in stem and leaf tissues of both PD-resistant and -susceptible grapevines was detected at different stages of growth and disease development in response to *Xf *infection using a pair of specific primers P450F1 and P450R1 (Table1) for the *CYP736B *coding region. Quantitative Real-time PCR analysis results showed that expression of *CYP736B *was much higher in leaf tissues than in stem tissues of both PD-resistant and -susceptible grapevines, ranging from 30 - 138.6 fold greater depending on growth stage (Table 2). In stem tissues of control PD-resistant plants, *CYP736B *expression at weeks 1 and 10 was similar but slightly lower at week 6 when expression of *CYP736B *was elevated by a factor of 1.3 (p < 0.05). In contrast, expression of *CYP736B *was increased by factors of 5.3 (p < 0.01) and 2.9 (p < 0.01) in stem tissues of the control PD-susceptible grapevine at week 6 and week 10. However, *Xf *infection did not result in a significant change in *CYP736B *expression in stem tissues of the PD-susceptible grapevine at week 1. Infection did cause a significant decrease of *CYP736B *expression in stem tissues of PD-susceptible grapevine to 4.08-fold less (p < 0.01) and 1.71-fold less (p < 0.05) at weeks 6 and 10, respectively. Interestingly, *CYP736B *expression was elevated up to 112.9-fold and 74.9-fold in leaf tissues relative to stem tissues of both control PD-resistant and -susceptible grapevines at week 1 post-inoculation, respectively, but down-regulated in leaf tissues with progression of plant growth under normal conditions without *Xf *infection. For example, *CYP736B *expression was decreased by 1.31-fold (p < 0.01) and 2.29-fold (p < 0.01) at weeks 6 and 10 in leaf tissues of the PD-resistant grapevine, whereas *CYP736B *expression decreased by 1.79-fold (p < 0.01) and 2.53-fold (p < 0.01) at weeks 6 and 10 in leaf tissues of the PD-susceptible grapevine. However, *Xf *infection resulted in increase of *CYP736B *expression up to 1.6-fold (P < 0.01) in leaf tissues of the PD-resistant grapevine and a decrease of *CYP736B *expression by 2.11-fold (P < 0.01) in leaf tissues of the PD-susceptible grapevine.

**Table 2:** Quantitative analyses of *CYP736B *gene expression in stem and leaf tissues of grapevines at different stages of growth (1week, 6 week and 10 week after inoculation) with *Xf *infection (T) and without *Xf *infection (C)

Tissue	Genotype	Growth Stage	Treatment	Relative Expression	Fold Difference in Expression Relative Folds for	Fold Difference in Expression
				Level (SD)	Growth Stages (p value)	*Xf *Infection (p value)
Stems	9621-67	1W	C	1 (reference)	1.00 (reference)	
			T	1.5 (0.56)		1.50 (0.02348)
		6W	C	1.3 (0.26)	1.30 (0.01938)	
			T	2.5 (1.00)		1.92 (0.00797)
		10W	C	0.8 (0.31)	0.80 (0.06844)	
			T	0.4 (0.26)		-2.00 (0.02928)
						
	9621-94	1W	C	1.1 (0.26)	1.1 (0.239829)	
			T	1.2 (0.41)		1.09 (0.26111)
		6W	C	5.3 (1.26)	5.30 (0.00000)	
			T	1.3 (0.37)		-4.08 (0.00001)
		10W	C	2.9 (0.98)	2.90 (0.00038)	
			T	1.7 (0.55)		-1.71 (0.01387)
						
Leaves	9621-67	1W	C	112.9 (19.56)	1.00 (reference)	
			T	94.8 (16.41)		-1.19 (0.05631)
		6W	C	86.4 (10.97)	-1.31 (0.00794)	
			T	138.6 (6.1)		1.60 (0.00000)
		10W	C	49.4 (15.43)	-2.29 (0.00005)	
			T	41 (14.26)		-1.20 (0.17655)
						
	9621-94	1W	C	74.9 (11.46)	-1.51 (0.00107)	
			T	70.2 (16.71)		-1.07 (0.28799)
		6W	C	63.2 (10.77)	-1.79 (0.00014)	
			T	30 (10.93)		-2.11 (0.00017)
		10W	C	44.6 (10.79)	-2.53 (0.00001)	
			T	40.9 (6.14)		-1.09 (0.24242)

The cDNA sequences of *CYP736B *transcripts from leaf samples of both PD-resistant and -susceptible genotypes with or without *Xf *infection were determined. The results indicated that amino acid sequences encoded by *CYP736B *transcripts were highly similar (99% identical) within each PD-resistant and -susceptible sampling group and between the two groups, suggesting that involvement of *CYP736B *in the defense response against *Xf *infection could be regulated at transcriptional and posttranscriptional levels.

### Pre-mRNA splicing patterns of CYP736B genes

The cDNA sequences of *CYP736B *transcripts from leaf and stem tissue of both PD-resistant and -susceptible grapevines at different stages of growth and development with or without *Xf *infection were cloned using *CYP736B *coding region specific primers P450F1 and P450R1. These cDNA sequences were further examined to determine if gene structure and/or posttranscriptional processing patterns changed over time and tissues. The results showed that *CYP736B *transcripts were spliced with GT as a donor site and AG as an acceptor site within the intron sequence of *CYP736B *pre-mRNAs in both PD-resistant and -susceptible samples (Figure 2). To study the dynamic pattern of pre-mRNA splicing of *CYP736B*, we designed a pair of Real-time PCR primers, SYBR-P450F2 and SYBR-P450R2 (Table 1), for quantitative analysis of the relative frequency of unspliced *CYP736B *transcripts in leaf tissues during growth and development in response to *Xf *infection. As shown in Table 3, the relative frequency of unspliced *CYP736B *transcripts was 3 to 4-fold less in leaf tissues of control PD-resistant and -susceptible grapevines at week 1 when genomic DNA was used as a control reference for Real-time Q-PCR analysis. The results indicate that most *CYP736B *pre-mRNA transcripts were correctly spliced in leaf tissues of both control PD-resistant and -susceptible grapevines at week 1 in plants not inoculated with *Xf*. In comparison, relative frequency of unspliced *CYP736B *transcripts was significantly increased up to 16.22- and 4.51-fold greater in leaf tissues of control PD- resistant plants at week 6 and 10, respectively. In contrast, relative frequency of unspliced *CYP736B *transcripts was as low as 1.18- and 2.21-fold less in leaf tissues of control PD-susceptible plants at week 6 and 10, respectively. However, when the leaf tissues of the PD- resistant grapevines was inoculated the relative frequency of unspliced *CYP736B *transcripts decreased to as low as 1.79- and 1.95-fold at week 1 and 6 and then increased to 3.59-fold greater at week 10. However, when leaf tissues of the PD- susceptible grapevines were inoculated, the relative frequency of unspliced *CYP736B *transcripts increased to 2.73- and 358.95-fold greater at week 1 and 6, respectively, and then decreased to 1.23-fold less at week 10. Representative cDNA sequencing confirmed that both spliced and unspliced *CYP736B *transcripts were generated in both PD-resistant and -susceptible grapevines irrespective of *Xf *infection (data not shown).

**Table 3:** Relative frequencies of unspliced *CYP736B *transcripts in leaf tissues of PD-resistant (9621-67) and -susceptible (9621-94) grapevines at different stages of growth (weeks after inoculation) with or without *Xf *infection

Genotype	Growth Stage	Treatment	Unspliced transcripts	SD	Fold Difference for Growth Stage	Fold Difference for Xf Infection
			Fold Difference		(p value)	(p value)
			1.00*	0.00		
9621-67	1W	C	-3.22	0.10	1.00 (reference)	
		T	-5.88	0.14		-1.79 (0.117687)
	6W	C	5.03	1.85	**16.22 **(0.005751)	
		T	2.58	0.59		**-1.95 **(0.047095)
	10W	C	1.40	0.25	**4.51 **(0.001136)	
		T	5.02	1.17		**3.59 **(0.003156)
9621-94	1W	C	-4.00	0.08	0.81 (0.237776)	
		T	-7.14	0.01		**2.73 **(0.033004)
	6W	C	-2.70	0.03	1.18 (0.198382)	
		T	131.30	11.21		**358.95 **(0.00002)
	10W	C	-1.47	0.07	**2.21 **(0.002999)	
		T	-1.79	0.02		**-1.23 **(0.022641)

Genomic DNA was used as a reference for unspliced/spliced transcript analysis.

### Determination of candidate 5' termini of grape CYP736B transcripts

**Figure 3:**
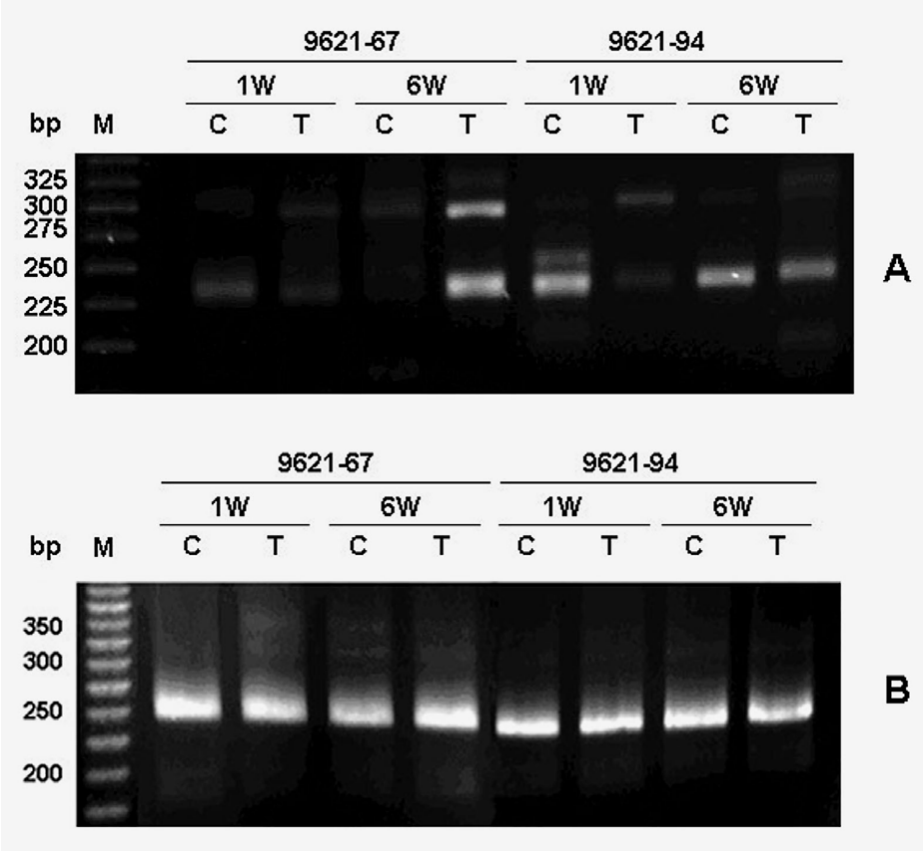
**A. 5'RACE display of *CYP736B *transcripts in both PD- resistant and susceptible grapevines in response to *Xf *infection**. **B**. 3'RACE display of *CYP736B *transcripts in both PD- resistant and susceptible grapevines in response to *Xf *infection. Names of grapevine genotype, sampling time are shown on the top, molecular sizes are shown on the left.

Due to lower levels of *CYP736B *expression in stem tissues at all growth stages analyzed and the most significant change of *CYP736B *gene expression in leaf tissues at week 6, the 5'RACE method was used to determine transcription initiation sites in leaf tissues of both PD-resistant and -susceptible grapevines at week 1 and 6 under inoculated and uninoculated conditions. As shown in Figure 3a, there were 1 - 3 major cDNA bands generated from leaf RNA templates from both PD-resistant and -susceptible grapevines at week 1 and week 6, with or without *Xf *inoculation. To elucidate the nature of these major multiple 5'RACE cDNA bands, we combined promoter and transcription start site prediction and experimental verification methods. Bioinformatics-aided promoter prediction revealed that there were potentially three major transcription start sites (Figure 2): TSS1 (-290 to -240), TSS2 (-136 to -86), and TSS3 (-69 to -19). 5'RACE cDNA cloning and sequencing analysis revealed that all of predicted transcription start sites were found in *CYP736B *transcripts as we predicted from the agarose gel analysis of the 3'RACE-amplified PCR products (Figure 3). We further quantified the relative abundance of major transcription start site usage using Real-time PCR analysis with a pair of primers SYBR-P450F1 and SYBR-P450R1. As summarized in Table 4, usage frequency of both TSS2 and TSS3 varied, from 0.66- to 9.20-fold for TSS2 and from 0.35- to 8.67-fold for TSS3, respectively, in leaf tissues of both PD -resistant and susceptible grapevines at different stages of growth without *Xf *infection. Compared to control plants, however, usage of TSS2 increased significantly in leaf tissues of resistant plants at week 1 (17.65-fold greater than susceptible control plants, 26.74-fold greater than resistant control plants, n = 3, p = 0.001854) and at the growth stage week 6 (37.12-fold greater than susceptible control plants, 4.03-fold greater than resistant control plants, n = 3, p = 0.017017) after *Xf *inoculation. In contrast, usage frequency of TSS2 increased significantly after *Xf *inoculation at week 1 (2.22-fold greater than control plants, n = 3, p = 0.003532) in leaf tissues of susceptible plants, but remained unchanged (1.42- and 1.56-fold for control and *Xf *infected plants, respectively, compared to the susceptible control plant for *Xf *infection at week 1, n = 3, p = 0.354589) at week 6 after *Xf *inoculation. Similarly, usage frequency of TSS3 remained unchanged at growth stage week 1 after *Xf *inoculation (0.62- and 0.50-fold, respectively compared to the susceptible control plants, n = 3, p = 0.204438), but greatly increased at the growth stage week 6 after *Xf *inoculation (8.67-fold higher than susceptible control plants, 24.77 fold higher than resistant control plants, n = 3, p = 0.000483) in leaf tissues of resistant plants. Usage frequencies of TSS3 were low in leaf tissues of susceptible plants at week 1 (0.4-fold compared to control plants, n = 3, p = 0.000203) and week 6 (0.23-fold compared to control plants, n = 3, p = 0.017818) after *Xf *inoculation.

**Table 4:** Relative abundance of transcription start site usage in TSS2 and TSS3 regions of *CYP736B *gene in leaf tissues of both PD-resistant (9621-67) and -susceptible (9621-94) plants after 1 and 6 weeks of inoculation with *Xf*

Genotype	Weeks	Treatment	TSS2			TSS3		
			
			Average	SD	t-test (p)	Average	SD	t-test (p)
			(Fold difference)			(Fold difference)		
9621-67	1	Control	0.66	0.36		0.62	0.19	
		*Xf*-Infected	17.65**	4.83	0.001854	0.50	0.15	0.204438
	6	Control	9.20	4.26		0.35	0.11	
		*Xf *-Infected	37.12*	14.68	0.017017	8.67**	1.66	0.000483
								
9621-94	1	Control	1.00	0.00		1.00	0.00	
		*Xf *-Infected	2.22**	0.42	0.003532	0.40**	0.10	0.000203
	6	Control	1.56	0.43		0.60	0.20	
		*Xf *-Infected	1.42	0.41	0.354589	0.23*	0.02	0.017818

** Significant level at P < 0.01; * Significant level at P < 0.05.

### Determination of candidate 3' termini of grape CYP736B transcripts

**Figure 4:**
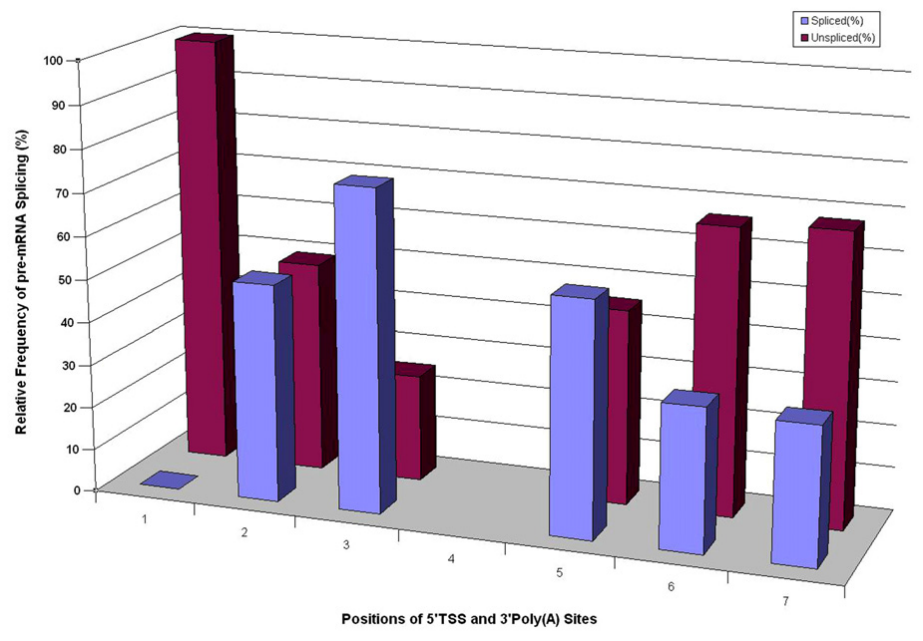
**Relationship between positions of 5'TSS/3'Poly(A) sites and *CYP736B *pre-mRNA splicing and the effect of transcription initiation and termination/polyadenylation on pre-mRNA splicing of *CYP736B *genes in leaf tissues of grapevines**. Three 5'TSS sites (left three) and three 3'Poly(A) sites (right three) are shown at the bottom, splicing frequency (column in blue) or unsplicing frequency (column in red) of *CYP736B *transcripts corresponding to each 5'TSS site and 3'Poly(A) site are shown on the left.

Since transcription termination involves polyadenylation, the 3'termini of *CYP736B *transcripts were cloned using 3'RACE method to determine termination and poly(A) signals of *CYP736B*. Several major cDNA bands with different intensities were obtained from leaf tissues of both PD- resistant and susceptible grapevines at week 1 and week 6 (Figure 4). To elucidate the nature of these major 3'RACE cDNA bands, we isolated and cloned each major 3'RACE cDNA. Sequence analysis revealed that there were three major polyadenylation sites, as predicted from the bioinformatics-aided polyadnylation analysis (Figure 2): poly(A)1 (++53 to ++58, AATAAT), poly(A)2 (++153 to ++159, AATTAAA), and poly(A)3 (++238 to ++245, AATAAA).

Based on results of 3'RACE cDNA display (Figure 4), it appears that Poly(A)1 was the major polyadenylation site utilized for *CYP736B *transcripts under all conditions tested, while Poly(A)2 and Poly(A)3 were less frequently used as alternative sites and subject to change upon plant growth and *Xf *inoculation. To quantify relative levels of representative 3'RACE cDNAs, usage frequency of *CYP736B *transcripts with Poly(A)2 was determined using Real-time PCR. In leaf tissues of PD- resistant plants after *Xf *inoculation, total usage frequency of Poly(A)2 site was increased 12.38-fold at week 1 (n = 3, p = 0.000057) but decreased 2.75-fold at week 6 (n = 3, p = 0.005304) when compared to respective control plants. However, usage frequency of Poly(A)2 in leaf tissues of susceptible plants was not significantly increased (1.28-fold greater than control, n = 3, p = 0.075134) upon *Xf *infection at week 1, and at week 6 (1.12-fold greater than control, n = 3, p = 0.414483) (Table 5).

**Table 5:** Relative abundance of poly(A)2 site in the 3' termini of *CYP736B *Transcripts in leaf tissues of both PD-resistant (9621-67) and -susceptible (9621-94) plants after 1 and 6 weeks of inoculation with *Xf*

Genotype	Weeks	Treatment	poly(A)2 Site		
			
			Average	SD	t-test (p)
			(Fold difference)		
9621-67	1	Control	1.49	0.39	
		*Xf *-Infected	18.44**	1.91	0.000057
	6	Control	37.35	8.40	
		*Xf *-Infected	13.6**	3.48	0.005304
					
9621-94	1	Control	1.00	0.00	
		*Xf *-Infected	1.28*	0.27	0.075134
	6	Control	1.17	0.23	
		*Xf *-Infected	1.12	0.26	0.414483

** Significant level at P < 0.01; * Significant level at P < 0.05.

### Correlation among transcription initiation, pre-mRNA splicing and polyadenylation of CYP736B genes

The objective of this study was to understand coordination of transcription initiation and termination of *CYP736B *and its impact on pre-mRNA splicing after *Xf *inoculation. First, we used a 5'UTR-defined long 3'RACE strategy to amplify full-length cDNAs of *CYP736B *transcripts with various 5' and 3' ends. Only two representative RNA samples were used in this test: PD-resistant and -susceptible leaf tissue harvested 6 weeks after *Xf *inoculation. The results revealed several distinguishable cDNA bands from each sample, as expected from 5' RACE and 3' RACE data (Figures 3a and 3b). Based on the 5' RACE and 3' RACE results and potential 5'TSS and 3'poly(A) site mapping results (Figures 2, Figure 3a and 3b), approximate size distributions of *CYP736B *transcripts with specific 5' and 3' termini were estimated, and corresponding cDNA bands excised from the gel for cloning. Then, we designed three upstream primers located at the three different 5'TSS regions: -300UF, -140UF, and -60UF (Table 1), and three downstream primers located at the three different 3'poly(A) regions: ++70UR, ++200UR, and ++260UR (Table 1). Different combinations of these primers were used to amplify full-length cDNA sequences of *CYP736B *transcripts with specific 5'TSS and 3'poly(A) termini. Finally, relative abundance of each *CYP736B *transcript with specific initiation and termination/polyadenylation sites were quantified by a Real-time PCR with primers SYBR-P450F1 and SYBR-P450R1. As shown in Table 6, relative abundance of *CYP736B *transcript in leaf tissues of resistant plants with a specific initiation site at the -300 position in the TSS1-containing region was increased as the distance of each transcription termination/polyadenylation site increased from poly(A)1 to poly(A)3 (P = 0.0000 ~ 0.2968, R^2 ^= 0.8940). Relative abundance of *CYP736B *transcript with a specific transcription initiation site at the -140 position in the TSS2-containing region also was increased as the distance of each transcription termination/polyadenylation site increased from poly(A)1 to poly(A)2, but not poly(A)3 (P = 0.0010 ~ 0.2828, R^2 ^= 0.8412). Relative abundance of *CYP736B *transcript with TSS2 sites was much higher than that with TSS1 sites in leaf tissues of PD- resistant plants. Relative abundance of *CYP736B *transcripts with the TSS1-containing region and TSS2-containing region in leaf tissues of susceptible plants showed similar patterns of change (P = 0.0000 ~ 0.4054, R^2 ^= 0.8450 within TSS1-containing region, P = 0.0030 ~ 0.3039, R^2 ^= 0.8936 within TSS2-containing region, and P = 0.0002 ~ 0.0018 between the TSS1-containing region and TSS2-containing region, respectively). However, abundance of each CYP736B transcript in susceptible plants was lower than in resistant plants (P = 0.0015 ~ 0.0875 as compared within TSS1-containing region; and P = 0.0082 ~ 0.3142 as compared within TSS2-containing region, respectively).

**Table 6:** Relative abundance of coordinated 5' TSS and 3' Poly(A) site usage for *CYP736B *gene transcription in leaf tissues of PD-resistant (9621-67) and -susceptible (9621-94) plants 6 weeks after *Xf *infection (fold difference)

Group	Genotype	From 5'TSS to 3'poly(A)	Mean	SD	P1¶		P2¶		**R**^**2 **^**§**
I	9621- 67	(-300) to (++70)	3.89	0.77			0.0001	0.0015	
		(-300) to (++200)	6.91*	1.77	0.0270		0.0003	0.0209	
		(-300) to (++260)	26.81**	2.73	0.0001	0.0002	0.0003	0.0384	0.8940
II	9621- 67	(-140) to (++70)	64.77	2.14				0.0082	
		(-140) to (++200)	77.24	11.99	0.0750			0.3142	
		(-140) to (++260)	166.21**	24.65	0.0013	0.0025		0.3017	0.8412
III	9621- 94	(-300) to (++70)	1.00	0.00			0.0002		
		(-300) to (++200)	2.68	1.74	0.0850		0.0018		
		(-300) to (++260)	20.34**	3.86	0.0001	0.0010	0.0010		0.8450
IV	9621- 94	(-140) to (++70)	46.91	7.47					
		(-140) to (++200)	85.16*	23.31	0.0270				
		(-140) to (++260)	153.03**	32.17	0.0031	0.0208			0.8936

¶ Student T-Test: P1 (within group) indicates the significance between two 5'TSS-3'Poly(A) types of transcripts within same assay group; P2 (between groups) indicates the significance between two 5'TSS-3'Poly(A) types of transcripts between groups.

§ R^2 ^shows correlation efficiency between each group of transcripts with fixed 5'TSS and various lengths of 3'-Poly(A) ends and their relative abundance (fold difference) within each assay group.

**Significant level at P < 0.01; * significant level at P < 0.0

We also cloned and sequenced representative cDNA sequences with different 5'TSS and 3'poly(A) sites of *CYP736B *transcripts from PD-resistant and -susceptible leaf tissues 6 weeks after *Xf *inoculation. The genomic DNA and cDNA sequence alignment indicated that pre-mRNA splicing patterns of *CYP736B *transcripts from PD-resistant and -susceptible leaf tissues 6 weeks after *Xf *inoculation remained the same as that in control plants even though various *CYP736B *transcripts contained different 5'TSS and 3'poly(A) sites (data not shown). Quantitative analysis of relative frequencies of each cDNA clone with or without intron sequence showed that the frequency of correct *CYP736B *pre-mRNA splicing was correlated with distance from transcription initiation sites to the translation start codon (ATG) or from transcription termination sites to the stop codon (TGA); the closer the distance, the higher the frequency of correctly spliced *CYP736B *pre-mRNA (Figure 3b). As distance from the poly(A)-containing region to the stop codon (TGA) increased, frequency of the spliced *CYP736B *RNA decreased. Statistical analysis showed that correlation of 5'UTRs to mRNA splicing was as high as R^2 ^= 0.8302 in resistant plants and R^2 ^= 0.9808 in susceptible plants. The correlation efficiency of 3'UTRs to mRNA splicing was as high as R^2 ^= 0.8922 in resistant plants and R^2 ^= 0.8678 in susceptible plants.

## Discussion

### CYP736B expression is regulated by Xf infection and development

Cytochrome P450 monooxygenase genes encode for a superfamily of enzymes in plants that seem to be involved in response to abiotic and biotic stresses [8,15]. Although several P450 genes play crucial roles in biosynthesis of a variety of endogenous lipophilic and antioxidative compounds, little is known about cytochrome P450 monooxygenase gene induction and regulation in response to pathogens. A previous study using cDNA microarray screening revealed that expression of several cytochrome P450 monooxygenase genes was tightly regulated during growth and development of grapevines in response to *X. fastidiosa *infection [unpublished data]. The current study identified a cluster of three cytochrome P450 monooxygenase genes (*CYP736A*, *CYP736B*, and *CYP736C*) that are organized as tandem repeats and flanked upstream with a RE-LTR sequence on chromosome 7. This organization is likely the result of gene duplication [16-18]. We found that *CYP736B *was expressed at very low levels in stem tissues but at higher levels in leaf tissues of both PD-resistant and -susceptible grapevines with or without *Xf *infection. While the level of *CYP736B *expression was quite different between leaf and stem tissues, patterns of gene expression in leaf and stem tissues were similar in both PD- resistant and susceptible grapevines. Differential expression in response to *Xf *infection between genotypes suggested that *CYP736B *may be involved in the host response to *Xf *infection. It is known that the relative density of *Xf *populations in stem tissues are much lower in PD-resistant compared to -susceptible grapevines [10]. This phenomenon may indicate that defense response genes, such as cytochrome P450 monooxygenase genes, may contribute to resistance against *Xf *infection in PD- resistant grapevines. In fact, a wide range of cytochrome P450 monooxygenases mediate biosynthesis of lignins, terpenes, alkaloids, and a variety of other secondary compounds. Various biochemical pathways are involved in this process: the DIMBOA biosynthesis pathway that is initiated in response to wounding and naphthalic anhydride treatment [4]; the camalexin biosynthesis pathway that is coordinately induced and strictly localized to the site of pathogen infection [19], and the lignin biosynthesis pathway that is induced to ward off passive microbial and fungal invaders or to lignify tissues to limit extent of damage caused by active invaders [20]. Cytochrome P450 monooxygenases have other functions, including versatile biocatalytic reactions that mediate primary detoxification of natural and synthetic toxins [20,11,21]. However, relationships between these biochemical pathways and genes regulating disease resistance in plants are largely unknown.

In this study, transcription and pre-mRNA splicing patterns of *CYP736B *changed differently, depending on growth stage and *Xf *infection status. Induced expression levels of *CYP736B *were correlated positively with resistance and negatively with susceptibility in both stem and leaf tissues 6 weeks post-inoculation. These results support the conclusion that *CYP736B *is involved in the defense response against *Xf *infection in grapevines. We further demonstrated that expression of *CYP736B *was post-transcriptionally regulated via pre-mRNA splicing. The very low frequency of unspliced *CYP736B *transcripts in leaf tissues of PD-resistant grapevines and the very high frequency of unspliced *CYP736B *transcripts in leaf tissues of PD- susceptible grapevines (especially at 6 weeks post-*Xf *inoculation) may reveal an important aspect of the grapevine *Xf *interaction; correctly spliced *CYP736B *transcripts would be functional in PD-resistant grapevines, whereas unspliced *CYP736B *transcripts would not be functional in PD- susceptible grapevines. Differences in *CYP736B *expression levels between stems and leaves of grapevines would suggest that the levels of monooxygenase biosynthesis also vary. It seems that *CYP736B *expression involves dynamic regulatory mechanisms at both transcription initiation and the post-transcription modification levels via a developmental-dependent splicing pathway in PD- resistant grapevines. This result provides the first evidence that *CYP736B *is involved in the defense response at a specific stage of pathogenesis in grapevines.

### Multiple transcription initiations contribute to the regulation of CYP736B gene expression

Transcriptional initiation of mRNAs is preceded by formation of a pre-initiation complex in eukaryotic cells. Diverse transcriptional initiation sites have been discovered by large scale mapping of mRNA start sites in the human genome [22]. Use of alternative transcription initiation sites is not uncommon in many animal and plant species [23,24]. Utilization of multiple transcription initiation sites may contribute to genetic flexibility in which expression may be temporally and spatially regulated. Furthermore, expression may be affected post-transcriptionally as demonstrated by differential expression and subcellular targeting of glutathione S-transferase F8 gene in *Arabidopsis *[25]. At present, regulatory mechanisms of cytochrome P450 monooxygenase genes in plants remain largely unknown. A previous study of cytochrome P450 expression and crosstalk in *Arabidopsis *revealed that most cytochrome P450 promoters contain the recognition sites MYB and MYC, an ACGT-core sequence, and TGA and W-boxes for WRKY transcription factors [8]. Upon identification of genomic organization and expression patterns in grapevines, further determination of transcriptional initiation sites became a key step towards understanding transcriptional regulatory mechanisms. This study identified three major transcription start sites. Usage frequencies of each transcription start site varied depending on plant genotypes, developmental stages, tissues, and pathogen infection. Transcription start sites TSS2 and TSS3 were the major targets of transcriptional regulation during grapevine growth and development. Our study revealed that two TATA boxes, one P-box and one I-box were located at the far upstream region of *CYP736B *transcripts, whereas two CAAT boxes and a G-box were located at the TSS1-containing region, and one W-box was located at the TSS2-containing region. Previous studies indicated that TATA boxes are the most common regulatory elements found in promoters of eukaryotic genes because they are associated with basal transcription initiation by RNA polymerase II, especially with *cis*-acting elements that enhance or repress transcription [26]. I-box (core sequence GATAA) and P-box (core sequence TGTAAAG) were associated with light and ABA responsiveness in tobacco and *Arabidopsis *respectively [27,28]. CAAT-box and G-box (core sequence CACGTG) were associated with tissue-specific regulation and heat shock induction in pea [29,30]. The W-box (core sequence CTGACT) is probably involved in elicitor- and wounding- responsive transcription of defense genes in tobacco [31,32]. If these *cis*-elements located within each *CYP736B *transcript are involved in physical interaction of the transcript with corresponding transcription factors, variation in frequencies of each transcription initiation site could reflect differences in *CYP736B *regulatory mechanisms triggered by *Xf*. If so, selective use of transcription initiation sites would play an important role in regulation of *CYP736B *expression in response to *Xf *infection.

### Polyadenylation sites determine transcriptional termination of CYP736B

Transcriptional termination by RNA polymerase II is known to be controlled by various regulatory elements located in 3'UTRs. Termination of transcription requires a functional polyadenylation site and the addition of a 3' poly(A) tail by poly(A) polymerase immediately after transcription [33,34]. The 3' end processing machinery functions to select an optimal poly(A) site between the poly(A) signal and a U-rich downstream element [35]. The sequence of the poly(A) signal and the number of uridine residues are known to affect polyadenylation efficiency [35,36]. However, the poly(A) site does not follow a strict consensus [37], even though a GC dinucleotide functions less efficiently than CA *in vitro *[38].

The research presented here revealed multiple poly(A) signals and multiple polyadenylation sites at the 3' UTR of *CYP736B *transcripts in both PD-resistant and -susceptible grapevines. There were 1 to 3 major polyadenylation sites with A or U as the terminal acceptor for polyadenylation. This result suggests that A and U are targets by poly(A) polymerase to synthesize poly(A) tails of *CYP736B *transcripts. Although there are examples of polyadenylation sites located between the poly(A) signal and a potential U-rich downstream element, a poly(A) signal could determine the optimal cleavage sites for *CYP736B *pre-mRNA polyadenylation. One possibility could be that the poly(A) polymerase-centered polyadenylation complex functions within the branched 3'UTR structure of *CYP736B *transcripts with some degree of flexibility after co-activation by poly(A) signals in grapevines. Given that total usage frequency of poly(A)2 and poly(A)3 sites changed differently between PD- resistant and susceptible grapevines after *Xf *inoculation, it is possible that bacterial virulence factors are involved directly or indirectly in modulation of *CYP736B *transcript polyadenylation in grapevines.

### Coordination of transcription initiation and polyadenylation play a role in CYP736B splicing

Transcription initiation, capping, cleavage/polyadenylation and pre-mRNA splicing are complex processes and tightly coupled to RNA polymerase II transcription. Transcription by RNA polymerase II and pre-mRNA processing are coordinated within the nucleus [39]. In general, capping at the 5' ends minimizes mRNA degradation and most importantly permits interaction with ribosomes. The 3' end is completed by the addition of a polyA tail, resulting in increased mRNA stability, and introns are removed at some steps during this process [39]. Bucheli et al. [40] found that the balanced competition of transcription factors with mRNA processing factors may promote recognition of proper polyadenylation sites while suppressing cryptic sites. Valencia et al. [41] suggested that a functional coupling is usually not essential for gene expression, but enhances the rate and/or efficiency of reactions that may serve to increase fidelity of gene expression in higher eukaryotes. Xin et al. [42] studied relationships between alternative promoters and pre-mRNA splicing and found that transcripts from different alternative promoters tended to splice differently. In the study presented here, variation in 5' transcription initiation sites or 3' polyadenylation sites affected *CYP736B *splicing. The combination of different transcription initiation sites and different 3' polyadenylation sites determined efficiency of *CYP736B *splicing in both PD- resistant and susceptible grapevines. For instance, transcription initiation from the TSS1-containing region always produced unspliced *CYP736B *RNAs in either PD- resistant or susceptible leaf tissues 6 weeks after *Xf *inoculation. As distance from the TSS-containing region to the start codon ATG decreased, frequency of spliced *CYP736B *RNA increased. Similarly, transcription termination at the poly(A)1 region of the 3'UTR always produced the highest frequency of spliced *CYP736B *RNAs. More importantly, frequency of correct *CYP736B *pre-mRNA splicing was correlated with both transcription initiation and termination sites distant to translation start and stop codons. The closer the distance from transcription initiation site to translation start codon and the distance from poly(A) site to translation stop codon, the higher the frequency of correctly spliced *CYP736B *transcripts. These relationships seem to be determined by changes in free energy within both 5'UTR and 3'UTR sequence-confined 2D structure constraints. Thus, our study revealed that both transcription initiation and termination have significant effects not only on relative abundance of *CYP736B *transcripts, but also on pre-mRNA splicing. Grapevine genotype/*Xf *interactions and their effect on *CYP736B *expression could be regulated by selective usage of transcription initiation and polyadenylation sites. The detailed regulatory mechanism of how *Xf *infection and grapevine/bacterial interactions affect selection of transcription initiation sites, polyadenylation sites, and pre-mRNA splicing remains to be understood. This report provides evidence that the cytochrome P450 monooxygenase *CYP736B *gene is involved in defense response at a specific stage of *Xf *infection in grapevines; multiple transcription initiation and polyadenylation sites exist for *CYP736B *in grapevine; and coordinated and selective use of transcription initiation and polyadenylation sites play an important role in regulation of *CYP736B *expression during growth, development and response to *Xf *infection.

## Methods

### Plant growth and treatment with Xf

The PD-resistant 9621-67 and -susceptible 9621-94 lines were selected from the 9621 population (D8909-15 (*V. rupestris *'A. de Serres × *V. arizonica *b42-26) × F8909-17 (*V. rupestris *'A. de Serres' × *V. arizonica/candicans *b43-17)) [11]. A total of sixty plants from each genotype were propagated and grown at 24 to 32°C in a greenhouse with day lengths adjusted to 18 hours. After one month of growth, plants were cut back to two buds and re-grown for about 6 weeks before inoculation. Plants were inoculated with 10 μl of the "Stag's Leap" strain of *Xf *(1 × 10^8 ^cfu/ml) as treatment groups or inoculated with 10 μl of *Xf*-free PW3 liquid media as control groups on the stem 10 cm above the pot surface following the procedure of Krivanek et al [10]. Each experimental unit consisted of three plants and was repeated three times in both treatment groups at each sampling date. Plants were arranged in a completely randomized design on greenhouse benches.

### Sample collection and evaluation

Leaf and stem tissues of three plants per treatment were collected at week 1, 6 and 10 after inoculation with or without *Xf*, respectively. Collected tissues were immediately placed in liquid nitrogen and stored at - 80°C until use. Symptoms were recorded as they appeared over a 12 week period on a separate set of 3 inoculated and 3 non-inoculated plants for each genotype grown under the same conditions.

### Gene identification and primer design

Based on the previous report [14], a genomic DNA sequence coding for a specific cytochrome P450 gene was identified from a grape genomic database (GenBank Accessions: AM475392.1, CAAP02000243.1, CAAP02005006.1) using the BLAST http://www.ncbi.nlm.nih.gov/ and FGENESH http://www.softberry.com/berry.phtml programs. A pair of primers, P450F1 and P450R1 (Table 1), was designed using Primer 3 Program http://biotools.umassmed.edu/bioapps/primer3_www.cgi to clone this gene. Another pair of primers, P450F2 and P450R2, for quantitative Real-time PCR analysis of this cytochrome P450 gene expression, was designed using GeneFisher Program http://bibiserv.techfak.uni-bielefeld.de/genefisher2/. Two pairs of primers, 5'UTRF1 and 5'UTRR1, 3'UTRF1 and 3'UTRR1 (Table 1), were designed using the Primer 3 Program to amplify and clone the 1,000 bp upstream and 600 bp downstream of the cytochrome P450 gene, respectively (Table 1).

### Total RNA isolation, cDNA synthesis and RT-PCR analysis of gene expression

Total RNA was isolated using Trizol RNA Isolation Kit (Invitrogen, Carlsbad, CA). First strand cDNA was synthesized using oligo(dT)_20 _primers with the cDNA Synthesis Kit (Invitrogen, Carlsbad, CA). RT-PCR analysis of gene expression was completed in a 25 μl of reaction that consisted of 1× reaction buffer (with 1.5 mM MgCl_2_), 2 mM dNTPs, 100 ng of cDNA template, 40 ng of primers, and 0.2 μl of Ampli Taq Gold DNA polymerase (5U/μl) (Applied Biosystems, Foster City, CA). The RT-PCR program consisted of preheating at 94°C for 5 min, 30 cycles of 94°C for 30 sec, 55°C for 30 sec, and 72°C for 3 min followed by a 72°C extension for 10 min. RT-PCR products were analyzed on a 1.2% of agarose gel in 1× TBE buffer with ethidium bromide staining. Real-time quantitative PCR analysis of gene expression was completed in a 25 μl of reaction that consisted of 1× SYBR Green PCR Master Mix (Applied Biosystems, Foster City, CA), 100 ng of template cDNA and 0.3 μM of each primers SYBR-P450F1 and SYBR-P450R1 (Table 1). The Real-time quantitative PCR was performed on the Bio-Rad iQ5 Multicolor Real-time PCR Detection System (Bio-Rad, Hercules, CA) with the PCR program of 95°C 10 min, 40 cycles of 95°C 10 sec and 60°C 1 min, The Real-time PCR results were analyzed using a PCR product conversion and accumulation law-based delta Ct method [43,44]. The same Real-time quantitative PCR also was used for quantitative analysis of pre-mRNA splicing with a pair of primers (SYBR-P450F2 and SYBR-P450R2) under the same experimental and analytic conditions (Table 1).

### Northern blot and hybridization

The Northern Max Formaldehyde-Based System for Northern Blots (Ambion, Texas) was used in this study according to the manufacturer's instruction. A full-length cDNA of grape cytochrome P450 monooxygenase gene CYP736B was labeled with biotin-dUTP at room temperature and used as probe for hybridization at 42°C for 18 hrs. Hybridization signals were detected using the BrightStar BioDetect Nonisotopic Detection Kit (Ambion, Texas).

### 5'RACE and 3'RACE Analyses

Determination of 5' upstream transcription start regions and 3' poly(A) termination signal regions were done with the 5'RACE and 3'RACE Kits (Invitrogen, Carlsbad, CA). The two gene specific primers used for upstream 5'RACE cDNA cloning were P450-5'RACE GSP2 and P450-5'RACE GSP3 (Table 1). The two gene specific primers used for downstream 3'RACE cDNA cloning were P450-3'RACE GSP1 and P450-3'RACE GSP2 (Table 1). Further quantitative measurements of the major 5'RACE and 3'RACE cDNAs were done using Real-time PCR analysis with primers SYBR-P450B 5'UTRF1 and SYBR-P450B 5'UTRR1, and SYBR-P450B 3'UTRF1 and SYBR-P450B 3'UTRR1, respectively, as described above (Table 1).

### Primer Extension Analysis

Two gene specific primers, P450-5'RACE GSP3 for 5' primer extension analysis and P450-3'RACE GSP2 for 3'primer extension analysis, were designed to determine upstream transcriptional initiation regions and downstream transcriptional termination regions of grape P450B transcripts, respectively (Table 1). Total RNA-based or the 5'RACE and 3'RACE cDNA-based primer extension was completed in a 25 μl reaction using a ThermoScript RT-PCR Kit (Invitrogen, Carlsbad, CA) according to the manufacturer's protocol. Primer extension products were separated in a 4% NuSieve^® ^3:1 Agarose gel (Lonza Rockland, Inc., Rockland, ME), transferred to a Nylon membrane, and detected by the BrightStar BioDetect Nonisotopic Detection Kit (Ambion, Texas).

### Quantification and cloning of representative full-length cDNA

To determine relationships among different transcription initiation sites, pre-mRNA splicing, and polyadenylation signals, a modified 5'UTR-defined long 3'RACE method was designed in combination of the representative specific 5'UTR primers with the standard 3'RACE technology (Invitrogen, Carlsbad, CA). These specific 5'UTR primers were -300UF, -140UF, and -60UF (Table 1). The 5'UTR-defined long 3'RACE cDNA products were electrophoresed in a 4% agarose gel with TBE Buffer. Similarly, three representative specific 3'UTR reverse primers also were designed from the standard 3'RACE sequences: ++70UR, ++200UR, and ++260UR (Table 1). The PCR system consisted of 1× LA Polymerase Buffer (with 1.5 mM MgCl_2_), 2 mM dNTPs, 100 ng of cDNA template, 40 ng of primers, and 0.125 μl of TAKARA *Taq *polymerase (1U/μl) in a 25 μl reaction. The PCR program consisted of preheating at 98°C for 10 sec, 30 cycles of 98°C for 10 sec, 55°C for 30 sec, and 72°C for 5 min, and post-PCR extension at 72°C for 10 min. The relative abundance of each specific 5'UTR and 3'UTR primer-defined P450 transcript was measured using Real-time Quantitative PCR as described above.

### DNA Cloning and Sequencing

Genomic DNA, cDNA and RT-PCR product bands were excised from agarose gels, purified with the PCR Gel DNA Purification Kit (Invitrogen, Carlsbad, CA), and cloned into pGEM-T easy vector (Promega, Madison, WI). Cloned DNA fragments were sequenced with M13F, M13R and a pair of internal P450 primers, P450F3 and P450R3 (Table 1), using BigDye Terminator V3.1 Sequencing and Clean-Up Kits (Applied Biosystems, Foster City, CA).

### 5'- and 3'-UTR DNA cis-acting regulatory element analysis

A PLACE database http://www.dna.affrc.go.jp/PLACE/signalscan.html was scanned to search key *cis*-acting regulatory elements located at the 5'- and 3'-UTRs of grape P450 genes. A PromScan program was used for grape P450 gene promoter analysis http://molbiol-tools.ca/promscan/.

### Protein Block analysis

The amino acid sequences of *in silico *translated P450 proteins were subjected to protein block analysis using the on-line program http://blocks.fhcrc.org/blocks/blocks_search.html.

### Statistical Analysis

Gene expression experiments in this study were repeated three times with three plants of each genotype at each time of sample collection. The statistic significance of experimental variation was calculated using ANOVA at confidence levels of 99% (*p *< 0.01) and 95% (*p *< 0.05). The Student T-test was used to analyze significance between treatments for gene expression at the same confidence levels of 99% (*p *< 0.01) and 95% (*p *< 0.05).

## Authors' contributions

DC, HL, YT and AW designed and conducted research experiments. DC was involved most expression analyses and the manuscript preparation. HL, AW, EC and DS were involved in organization of the work, writing and editing of the manuscript. HL was PI and initiator of the project. All authors took part in drafting, reviewing and approval of the final manuscript.

## References

[B1] Isin EM Guengerich FP Complex reactions catalyzed by cytochrome P450 enzymes Biochimica Biophysica Acta 2007 1770 314 329 10.1016/j.bbagen.2006.07.003 17239540

[B2] Chou WM Kutchan T Enzymatic oxidation in the biosynthesis of complex alkaloids Plant J 1998 15 289 300 10.1046/j.1365-313X.1998.00220.x 9750342

[B3] Durst F O'Keefe DP Plant cytochrome P450: an overview Drug Metabol Drug Interact 1995 12 171 187 8820851 10.1515/dmdi.1995.12.3-4.171

[B4] Persans MW Wang J Schuler M Characterization of maize cytochrome P450 monooxygenases induced in response to safeners and bacterial pathogens Plant Physiol 2001 125 1126 1138 10.1104/pp.125.2.1126 11161067 PMC64911

[B5] Schuhegger R Nafisi M Mansourova M Peterson BL Olsen XE Svatos A Halkier BA Glawischnig E CYP71B15 (PAD3) catalyzes the final step in camalexin biosynthesis Plant Physiol 2006 141 1248 1254 10.1104/pp.106.082024 16766671 PMC1533948

[B6] Glazebrook J Contrasting mechanisms of defense against biotrophic and necrotrophic pathogens Annu Rev Phytopathol 2005 43 205 227 10.1146/annurev.phyto.43.040204.135923 16078883

[B7] Kliebenstein DJ Rowe HC Denby KJ Secondary metabolites influence Arabidopsis/Botrytis interactions: variation in host production and pathogen sensitivity Plant J 2005 44 25 36 10.1111/j.1365-313X.2005.02508.x 16167893

[B8] Narusaka Y Narusaka M Seki M Umezawa T Ishida J Nakajima M Enju A Shinozaki K Crosstalk in the responses to abiotic and biotic stresses in Arabidopsis: analysis of gene expression in cytochrome P450 gene superfamily by cDNA microarray Plant Mol Biol 2004 55 327 342 10.1007/s11103-004-0685-1 15604685

[B9] Almeida RPP Purcell AH Transmission of *Xylella fastidiosa *to grapevines by *Homalodisca coagulata *(Hemiptera: Cicadellidae) J Econ Entomol 2003 96 264 271 10.1603/0022-0493-96.2.264 14994789

[B10] Krivanek AF Walker MA *Vitis *resistance to Pierce's disease is characterized by differential *Xylella fastidiosa *populations in stems and leaves Phytopathol 2005 95 44 52 10.1094/PHYTO-95-0044 18943835

[B11] Krivanek AF Famula TR Tenscher A Walker MA Inheritance of resistance to *Xylella fastidiosa *within a *Vitis rupestris *x *Vitis arizonica *hybrid population Theor Appl Genetics 2005 111 110 119 10.1007/s00122-005-1999-3 15864525

[B12] Therne ET Stevenson JF Rost TL Labavitch JM Matthews MA Pierce's disease symptoms: Comparison with symptoms of water deficit and the impact of water deficits Am J Enol Vitic 2006 57 1 11

[B13] Krivanek AF Riaz SN Walker MA Identification and molecular mapping of *PdR1*, a primary resistance gene to Pierce's disease in *Vitis* Theor Appl Genetics 2006 112 1125 1131 10.1007/s00122-006-0214-5 16435126

[B14] Lin H Doddapanneni H Takahashi Y Walker A Comparative analysis of ESTs involved in grape responses to *Xylella fastidiosa *infection BMC Plant Biol 2007 7 8 doi:10.1186/1471-2229-7-8 10.1186/1471-2229-7-8 17316447 PMC1821027

[B15] Ehlting J Sauveplane V Olry A Ginglinger J Provart NJ Werck-Reichhart D An extensive (co-)expression analysis tool for the cytochrome P450 superfamily in *Arabidopsis thaliana* BMV Plant Biol 2008 8 47 10.1186/1471-2229-8-47 PMC2383897 18433503

[B16] Cheng DW Armstrong KC Tinker N Wight CP He S Lybaert A Fedak G Molnar SJ Genetic and physical mapping of Lrk10-like receptor kinase sequences in hexaploid oat (*Avena sativa *L.) Genome 2002 45 100 9 10.1139/g01-135 11908651

[B17] Cheng DW Shan H Armstrong KC Modified expression and regulation of receptor kinase genes in response to rust pathogens in hexaploid oat Physiologic and Molecular Plant Pathol 2002 61 281 288 10.1006/pmpp.2003.0441

[B18] Cheng DW Armstrong KC Drouion G McElroy A Fedak G Molnar SD Isolation and identification of *Triticeae *chromosome 1 receptor-like kinase genes in diploid, tetraploid and hexaploid species of the genus *Avena* Genome 2003 46 119 127 10.1139/g02-111 12669804

[B19] Glawischnig E The role of cytochrome P450 enzymes in the biosynthesis of camalexin Biochem Soc Trans 2006 34 1206 1028 10.1042/BST0341206 17073786

[B20] Berenbaum MR Rosenthal G, Berenbaum M Herbivores: Their Interactions with Secondary Plant Metabolites 1991 1 Academic Press, San Diego 221 249

[B21] Werck-Reichhart D Hehn A Didierjean L Cytochromes P450 for engineering herbicides tolerance Trends Pharmacol Sci 2000 5 116 123 10.1016/S1360-1385(00)01567-3 10707077

[B22] Suzuki Y Taira H Tsunoda T Mizushima-Sugano J Sese J Hata H Ota T Isogai T Tanaka T Morishita S Diverse transcriptional initiation revealed by fine, large-scale mapping of mRNA start sites EMBO Rep 2001 2 388 393 11375929 10.1093/embo-reports/kve085 PMC1083880

[B23] Ayoubi TA Van De Ven WJ Regulation of gene expression by alternative promoters FASEB J 1996 10 453 460 8647344

[B24] Kuhn K Weihe B Borner T Multiple promoters are a common feature of mitochondrial genes in Arabidopsis Nucleic Acid Res 2005 33 337 346 10.1093/nar/gki179 PMC546163 15653634

[B25] Thatcher LF Carrie C Andersson CR Sivasithamparam K Whelan J Singh KB Differential gene expression and subcellular targeting of Arabidopsis glutathione S-transferase F8 is achieved through alternative transcription start sites J Biol Chem 2007 282 28915 28928 10.1074/jbc.M702207200 17670748

[B26] Bassett CL Nickerson ML Farrell RE Harrison M Multiple transcripts of a gene for a leucine-rich repeat receptor kinase from morning glory (*Ipmoea nil*) originate from different TATA boxes in a tissue-specific manner Mol Genet Genom 2004 271 752 760 10.1007/s00438-004-1031-7 15221460

[B27] Martinez-Hernandez A Lopez-Ochoa L Arguello-Astorga G Herrera-Estrella L Functional properties and regulatory complexity of a minimal RBCS light-responsive unit activated by phytochrome, cryptochrome and plastid signals Plant Physiol 2002 128 1223 1233 10.1104/pp.010678 11950971 PMC154250

[B28] Nakashima K Fujita Y Katsura K Maruyama K Narusaka Y Seki M Shinozaki K Yamaguchi-Shinozaki K Transcriptional regulation of ABI3- and ABA-responsive genes including RD29B and RD29A in seeds, germinating embryos, seedlings of Arabidopsis Plant Mol Biol 2006 60 51 68 10.1007/s11103-005-2418-5 16463099

[B29] Chandrasekharan MB Bishop KJ Hall TC Module-specific regulation of the beta-phaseolin promoter during embryogenesis Plant J 2003 33 853 866 10.1046/j.1365-313X.2003.01678.x 12609027

[B30] Shirsat A Wilford N Croy R Boulter D Sequences responsible for the tissue specific promoter activity of a pea legumin gene in tobacco Mol Gen Genet 1989 215 326 331 10.1007/BF00339737 2710102

[B31] Nishiuchi T Shinshi H Suzuki K Rapid and transient activation of transcription of the ERF3 gene by wounding in tobacco leaves: Possible involvement of NtWRKYs and autorepression J Biol Chem 2004 279 55355 55361 10.1074/jbc.M409674200 15509567

[B32] Yamamoto S Nakano T Suzuki K Shinshi H Elicitor-induced activation of transcription via W box-related cis-acting elements from a basic chitinase gene by WRKY transcription factors in tobacco Biochem Biophys Acta 2004 1679 279 287 15358520 10.1016/j.bbaexp.2004.07.005

[B33] Proudfoot NJ How RNA polymerase II terminates transcription in higher eukaryotes Trends Biochem Sci 1989 14 105 110 10.1016/0968-0004(89)90132-1 2658217

[B34] Wilusz CJ Mormington M Peltz SW The cap-to-tail guide to mRNA turnover Nature Rev 2001 2 237 246 10.1038/35067025 11283721

[B35] Chou ZF Chen F Wilusz J Sequence and position requirements for uridylate-rich downstream elements of polyadenylation signals Nucl Acids Res 1994 22 2525 2531 10.1093/nar/22.13.2525 7518915 PMC308205

[B36] Colgan DF Manley JL Mechanism and regulation of mRNA polyadenylation Genes Dev 1997 11 2755 2766 10.1101/gad.11.21.2755 9353246

[B37] Zhao J Hyman L Moore C Formation of mRNA 3' ends in eukaryotes: mechanism, regulation, and interrelationships with other steps in mRNA synthesis Microbio Mol Biol Rev 1999 63 404 445 10.1128/mmbr.63.2.405-445.1999 PMC98971 10357856

[B38] Chen F MacDonald CC Wilusz J Cleavage site determinants in the mammalian polyadenylation signal Nucl Acids Res 1995 23 2614 2620 10.1093/nar/23.14.2614 7651822 PMC307082

[B39] Cramer P Srebrow A Kadener S Werbajh S de la Mata M Melen G Nogues G Kornblihtt AR Coordination between transcription and pre-mRNA processing FEBS Lett 2001 498 179 182 10.1016/S0014-5793(01)02485-1 11412852

[B40] Bucheli ME He X Kaplan CD Moore CL Buratowski S Polyadenylation site choice in yeast is affected by competition between Npl3 and polyadenylation factor CFI RNA 2007 3 1756 1764 10.1261/rna.607207 PMC1986811 17684230

[B41] Valencia P Dias AP Reed R Splicing promotes rapid and efficient mRNA export in mammalian cells Proc Natl Acad Sci USA 2008 105 3386 3391 10.1073/pnas.0800250105 18287003 PMC2265164

[B42] Xin D Hu L Kong X Alternative promoters influence alternative splicing at the genomic level PLoS ONE 2008 3 e2377 10.1371/journal.pone.0002377 18560582 PMC2409967

[B43] Cheng DW Transformation and accumulation laws of PCR amplification products Hereditas (Beijing) 1991 13 33 38

[B44] Bookout AL Cummins CL Mangelsdorf DJ Pesola JM Kramer MF High-throughput real-time quantitative reverse transcription PCR Curr Proto Mol Biol 2006 Chapter 15 Unit 15.8 10.1002/0471142727.mb1508s73 18265376

